# Quantitative imaging parameters to predict the local staging of prostate cancer in intermediate- to high-risk patients

**DOI:** 10.1186/s13244-022-01217-4

**Published:** 2022-04-15

**Authors:** Riccardo Laudicella, Stephan Skawran, Daniela A. Ferraro, Urs J. Mühlematter, Alexander Maurer, Hannes Grünig, Hendrik J. Rüschoff, Niels Rupp, Olivio Donati, Daniel Eberli, Irene A. Burger

**Affiliations:** 1 Department of Nuclear Medicine, University Hospital Zurich, University of Zurich, Rämistrasse 10, 8091 Zürich, Switzerland; 2 Department of Biomedical and Dental Sciences and Morpho-Functional Imaging, Nuclear Medicine Unit, University of Messina, Messina, Italy; 3 Department of Radiology and Oncology, Faculdade de Medicina, FMUSP, Universidade de Sao Paulo, Sao Paulo, Brazil; 4 Department of Pathology, University Hospital Zürich, University of Zurich, Zurich, Switzerland; 5 Interventional and Diagnostic Radiology, University Hospital Zurich, University of Zurich, Zurich, Switzerland; 6 Department of Urology, University Hospital Zürich, University of Zurich, Zurich, Switzerland; 7 Department of Nuclear Medicine, Kantonsspital Baden, Baden, Switzerland

**Keywords:** Extracapsular extension, Seminal vesicle infiltration, PSMA PET (MRI) Prostate cancer, Prediction

## Abstract

**Objectives:**

PSMA PET/MRI showed the potential to increase the sensitivity for extraprostatic disease (EPD) assessment over mpMRI; however, the interreader variability for EPD is still high. Therefore, we aimed to assess whether quantitative PSMA and mpMRI imaging parameters could yield a more robust EPD prediction.

**Methods:**

We retrospectively evaluated PCa patients who underwent staging mpMRI and [^68^Ga]PSMA-PET, followed by radical prostatectomy at our institution between 01.02.2016 and 31.07.2019. Fifty-eight cases with PET/MRI and 15 cases with PET/CT were identified. EPD was determined on histopathology and correlated with quantitative PSMA and mpMRI parameters assessed by two readers: ADC (mm^2^/1000 s), longest capsular contact (LCC, mm), tumor volume (cm^3^), PSMA-SUV_max_ and volume-based parameters using a fixed threshold at SUV > 4 to delineate PSMA_total_ (g/ml) and PSMA_vol_ (cm^3^). The *t* test was used to compare means, Pearson’s test for categorical correlation, and ROC curve to determine the best cutoff. Interclass correlation (ICC) was performed for interreader agreement (95% CI).

**Results:**

Seventy-three patients were included (64.5 ± 6.0 years; PSA 14.4 ± 17.1 ng/ml), and 31 had EPD (42.5%). From mpMRI, only LCC reached significance (*p* = 0.005), while both volume-based PET parameters PSMA_total_ and PSMA_vol_ were significantly associated with EPD (*p* = 0.008 and *p* = 0.004, respectively). On ROC analysis, LCC, PSMA_total_, and PSMA_vol_ reached an AUC of 0.712 (*p* = 0.002), 0.709 (*p* = 0.002), and 0.718 (*p* = 0.002), respectively. ICC was moderate–good for LCC 0.727 (0.565–0.828) and excellent for PSMA_total_ and PSMA_vol_ with 0.944 (0.990–0.996) and 0.985 (0.976–0.991), respectively.

**Conclusions:**

Quantitative PSMA parameters have a similar potential as mpMRI LCC to predict EPD of PCa, with a significantly higher interreader agreement.

**Supplementary Information:**

The online version contains supplementary material available at 10.1186/s13244-022-01217-4.

## Key points

- Volume-based quantitative PSMA parameters (PSMAtotal and PSMAvol) are equally good to predict EPD compared to the longest capsular contact measured on mpMRI.
- The interreader agreement, however, is significantly higher for both PET parameters.

## Introduction

An accurate staging assessment is of paramount importance to best address therapeutical strategy and to improve patient’s outcome thus avoiding or minimizing potential side effects such as urinary incontinence and erectile dysfunction [1]. In the staging process of prostate cancer (PCa), attention must be paid to the presence of extraprostatic disease (EPD) in terms of extracapsular extension (ECE) and seminal vesicle infiltration (SVI), which determine a staging upgrade to T3 status [2]. ECE and SVI require essential considerations regarding peri-capsular structures such as neurovascular bundles before considering radical prostatectomy (RPE). Therefore, to improve RPE effectiveness, careful preoperative planning aiming at nerve-sparing surgery is essential, and high-risk PCa (≥ T2c or ISUP > 3) or presence of ECE is a contraindication to such a conservative approach. However, clinical evaluation of EPD is challenging and often false negative [3], and nomograms often give different cutoffs and do not include information about the location of EPD [4, 5]. The introduction of multiparametric magnetic resonance imaging (mpMRI) and its improvements (i.e., PIRADS 2.1 criteria [6]) increased the early detection of PCa and the accuracy of EPD prediction compared to clinical parameters [7]. However, interreader variability of mpMRI interpretation is still high [8]. Prostate-specific membrane antigen (PSMA) PET/CT was introduced in the PCa scenario for the assessment of biochemical recurrence disease (BCR), mainly due to its higher sensitivity and detection rate of disease compared to other radiotracers and mpMRI [3, 9]. Recently published results revealed higher management change, reduced equivocal findings, and 27% greater accuracy for PSMA PET/CT than conventional imaging regarding nodal and distant disease assessment, leading to additional rising interest for its use in PCa staging [10, 11]. Advanced refinements in the PCa molecular imaging scenario have been observed with the introduction of simultaneous PET/MRI scanners [12], being slightly more reproducible and sensitive than mpMRI (but faintly less specific) for the detection of ECE and/or SVI [13]. However, the interreader agreement remains suboptimal for both imaging modalities. Quantitative parameters are less subjective than visual analysis, and therefore, prediction of EPD based on such parameters is potentially more reliable. This study aimed to assess which quantitative imaging parameter of mpMRI and PSMA PET best predict EPD (pT3 status).

## Material and methods

### Patients

**Fig. 1:**
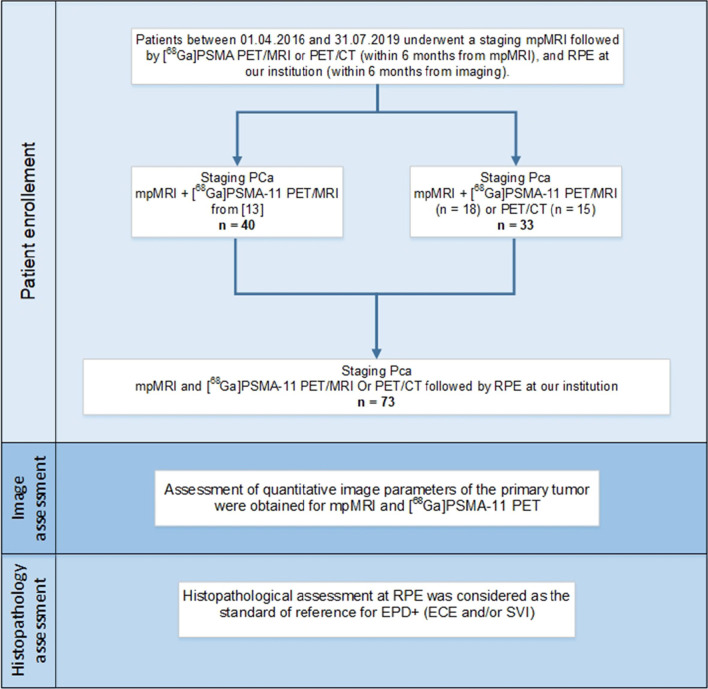
Patients’ selection and inclusion in the study

In this retrospective study, we included all the PCa patients who, between 01/04/2016 and 31/07/2019, underwent a staging mpMRI followed by [^68^Ga]PSMA PET/MRI or PET/CT (within 6 months from mpMRI) and RPE at our institution (within 6 months from imaging) performed at our institution using a robot-assisted trans-peritoneal laparoscopic approach with bilateral extended lymph nodal dissection (four-arm Da Vinci S system, Intuitive Surgical, Inc.). Patients were excluded, if the RPE was not performed in our institution within 6 months or is the patient refused the general consent for retrospective analysis of his data. The study cohort is based on the published analysis of staging mpMRI and [^68^Ga]PSMA-11 PET/MRI (*n* = 40) [13] and was extended to also include consecutive patients that after that underwent mpMRI and PET/MRI (*n* = 18) or PET/CT (*n* = 15) before RPE (Fig. 1) resulting in 58 cases with PET/MRI and 15 cases with PET/CT. The study was approved by the institutional review board, and all patients signed a general informed consent (2018–01284). Analysis was performed according to STARD 2015 guidelines [14].

### mpMRI

All patients underwent mpMRI for staging PCa with a 1.5 T or a 3.0 T at different institutions (55/73 in house, 18/73 external institutions) following current guidelines [6]. The mpMRI protocol consisted of three planes T2-weighted fast spin-echo images covering the prostate gland and the SV. Transverse diffusion-weighted imaging was performed. The apparent diffusion coefficient (ADC) maps were calculated by using three b values and an interpolated high-*b*-value image at 1400 s/mm^2^. Dynamic contrast material-enhanced MRI was performed to yield transverse sections with a temporal resolution of fewer than 8 s. Gadoterate meglumine (Dotarem; Guerbet) was used as a contrast agent in a dose of 0.1 mmol per kilogram of body weight at a 1.5 ml/s flow rate. The details for the acquired in-house mpMRI sequences are given in the Additional file 1: Supplemental Table 1.

### PET

Sixty minutes after [^68^Ga]PSMA-11 administration, 58/73 patients underwent PET/MRI (SIGNA PET/3.0 T MRI, GE Healthcare) and 15/73 PET/CT (Discovery VCT 690 or Discovery MI PET/CT, GE Healthcare) scans for staging PCa with an administered [^68^Ga]PSMA-11 dose of 2 MBq/kg (mean activity 132.9 ± 19.1 MBq, range 94–177). Scans were performed with the same protocol for prostate imaging as recently described [15]. To reduce the radiopharmaceutical activity in the urinary system, furosemide was injected intravenously 30 min before the tracer injection (0.13 mg/kg), and patients were asked to void before the scan. The institutional protocol was in agreement with the joint EANM-SNMMI procedure guidelines [16].

### Histopathology assessment—standard of reference

Pathological assessment of RPE specimens was considered as the standard of reference for EPD + (ECE and/or SVI). Histopathological analyses were performed by two experienced genito-urinary pathologists (NR, HJR) with 11 and 13 years of experience, respectively, following the WHO2016/ISUP prognostic grade groups guidelines [17].

### Image analysis

Quantitative image parameters of the primary tumor were obtained for mpMRI and [^68^Ga]PSMA-11 PET. For mpMRI readers acquired the average and minimum ADC values (ADC_mean_ and ADC_min_, mm^2^/1000 s), the longest capsular contact (LCC, mm), and the tumor volume (cm^3^). mpMRI LCC (mm) was defined as the maximum curvilinear length of PCa in contact with the prostatic capsule among all axial sections of the T2w-images in which the lesion was visible. On [^68^Ga]PSMA-11 PET readers quantified PSMA uptake with SUV_max_ and volume-based measures, using a fixed threshold at SUV > 4 to delineate total PSMA uptake (PSMA_tot_, g/ml), and PSMA volume (PSMA_vol_, cm^3^). All the PET/CT, PET/MRI, and mpMRI images were analyzed in a dedicated review workstation (Advantage Workstation, Version 4.6 or 4.7, GE Healthcare), which enables the review of the PET, CT, MRI, or mpMRI images side by side and in fused mode. mpMRI images were assessed by SS (Radiologist) and HG (Radiologist and Nuclear Medicine Physician) with 1, and 4 years of experience, respectively. PET/MRI and PET/CT images were assessed by RL and DAF (Nuclear medicine Physicians) with 2 and 2 years of experience, respectively.

### Statistical analysis

Statistical analyses were performed using SPSS statistics software, version 26 (IBM). Descriptive analyses were used to display patient data as mean and range; frequency distribution with percentages was used to summarize categorical variables, and means with standard deviations or medians were used to describe continuous variables. The t test was used to compare the mean values. The correlation between pT3 status (EPD+) and clinical (ISUP biopsy and ISUP RPE, cT staging, age, PSA) or imaging quantitative parameters (ADC_mean,_ ADC_min,_ mpMRI LCC and volume, SUV_max_, PSMA_tot_, and PSMA_vol_) were assessed with a 2-tailed Pearson correlation test. A *p*-value of less than 0.05 was considered statistically significant. The ability of the most significant clinical and imaging parameters to predict pT3 status (presence of ECE and/or SVI) was assessed with receiver operating characteristics (ROC) analysis and calculation of the area under the ROC curve (AUC). Statistical analyses were performed by RL, DAF and IAB (Nuclear Medicine Physicians). Finally, the two-way intraclass correlation coefficient (ICC) was used to measure interrater agreement for all significant quantitative parameters on mpMRI and PSMA PET. Based on the 95% confidence interval (CI) of the ICC estimate, values < 0.5, 0.5–0.75, 0.75–0.9, and > 0.90 were considered of poor, moderate, good, and excellent reliability, respectively [18]. Non-overlapping CI between ICC was considered as significantly different.

## Results

Seventy-three patients were identified, of those EPD (ECE or SVI) was present in 31 (42.5%), while 42/73 patients had no EPD (57.5%). Patient characteristics are summarized in Table 1.

**Table 1:** Patients’ characteristics (data are shown as mean ± SD)

	Total	EPD +	EPD-	*p*-value
Number of patients	73	31	42	
Age (years)	64.5 ± 6 (51–78)	66.3 ± 5.4 (53–76)	63.2 ± 6.2 (51–78)	**0.026**
PSA before PET/MRI (ng/ml)	14.4 ± 17.1 (1.2–104)	22 ± 20 (2.1–104)	10.3 ± 10.2 (1.22–55)	**0.031**
**MRI (*n*)**				
Internal–external	55–18	18–13	37–5	
1.5–3.0 T	2–71	1–30	1–41	
Endorectal coil–No coil	8–65	6–25	2–40	
**Time between mpMRI and PET**				
Days	64.6 ± 50.5 (0–175)	65.5 ± 48.1 (0–174)	63.9 ± 52.2 (0–175)	0.729
**Clinical T stage (*n*)**		0.052		
T1	46/73 (63%)	16/31 (52%)	30/42 (71.5%)	
T2	24/73 (32.5%)	12/31 (38.5%)	12/42 (28.5%)	
T3	2/73 (3%)	2/31(6.5%)	0/42 (0%)	
T4	1/73 (1.5%)	1/31 (3%)	0/42 (0%)	
**ISUP biopsy grade *n* (%)**		0.220		
1	2/73 (3%)	0/31 (0%)	2/42 (5%)	
2	10/73 (14%)	6/31 (19.5%)	4/42 (10%)	
3	15/73 (20%)	4/31 (13%)	11/42 (26%)	
4	30/73 (41%)	10/31 (32%)	20/42 (47%)	
5	16/73 (22%)	11/31 (35.5%)	5/42 (12%)	
**Time between PET and RPE**				
Days	38.3 ± 31.2 (1–177)	36.2 ± 23.2 (3–93)	39.9 ± 35.9 (1–177)	0.969
**ISUP RPE grade *n* (%)**				** < 0.001**
1	0/73 (0%)	0/31 (0%)	0/42 (0%)	
2	9/73 (12.5%)	3/31 (10%)	6/42 (14%)	
3	28/73 (38.5%)	5/31 (16%)	23/42 (55%)	
4	17/73 (23%)	8/31 (26%)	9/42 (21%)	
5	19/73 (26%)	15/31 (48%)	4/42 (10%)	

Statistically significant *p*-values are marked by bold font

EPD extraprostatic disease (on RPE specimen); SD standard deviation; mpMRI multiparametric magnetic resonance imaging; PET positron emission tomography/magnetic resonance imaging; PSA prostate-specific antigen; ISUP international society of urological pathology; and RPE radical prostatectomy

### Correlation between EPD and clinical parameters

**Fig. 2:**
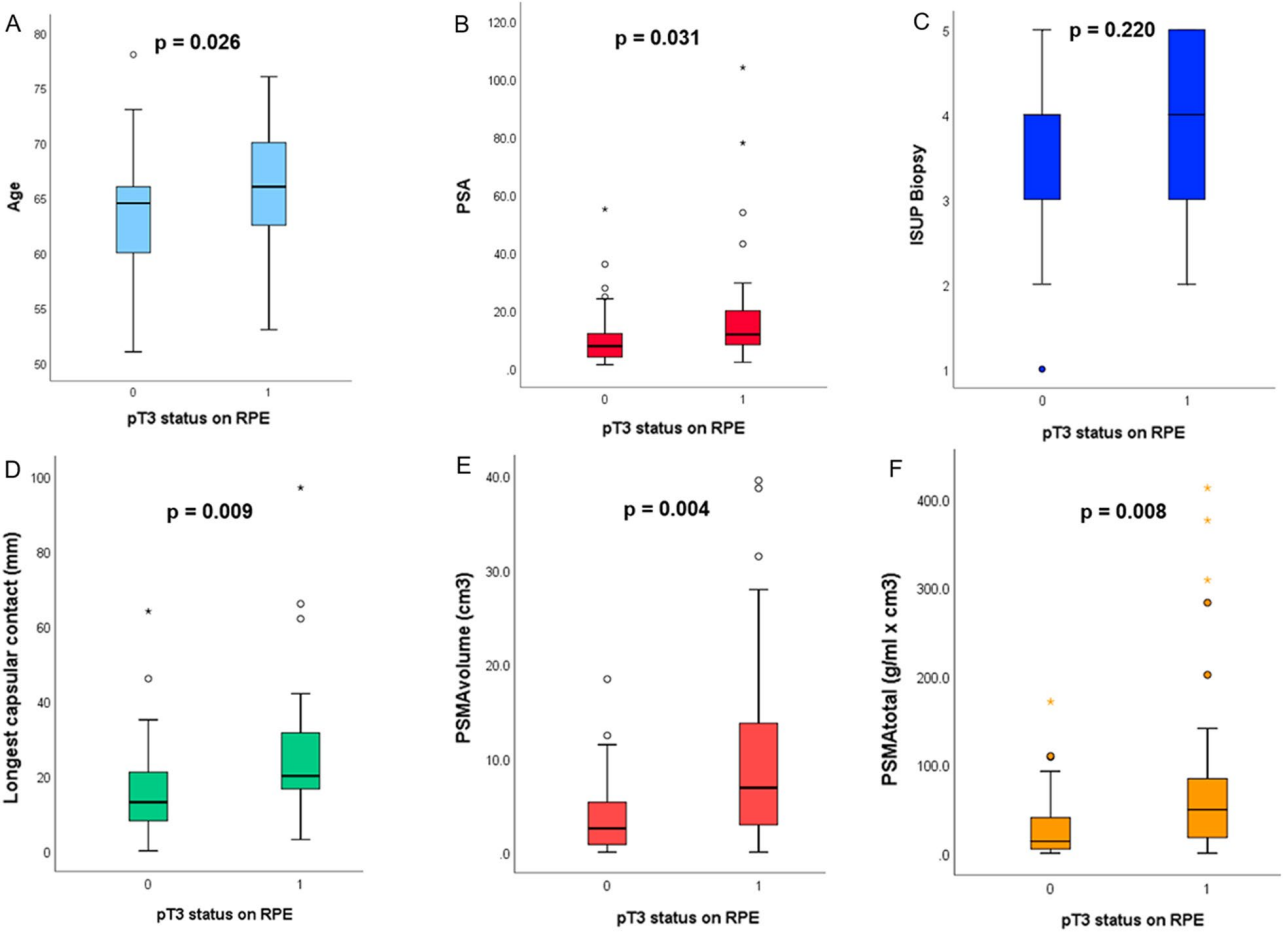
Box plots illustrating the relationship between pT3 status and (**a**) age, (**b**) PSA, (**c**) ISUP biopsy, (**d**) longest capsular contact, (**e**) PSMA_volume_, and (**f**) PSMA_total_

The mean and SD values for age, PSA, the timing between mpMRI, PET, and RPE are given in Table 1. Both age and PSA were significantly higher in the EPD group (*p* = 0.026 and *p* = 0.031, respectively). The biopsy-based ISUP grade and the clinical T-stage, however, did not correlate with EPD (*p* = 0.220 and *p* = 0.052, respectively) (Fig. 2a–c).

### Correlation between EPD and imaging parameters

The mean and SD values for all quantitative imaging parameters from mpMRI are given in Table 2. For LCC, the mean values between EPD-positive and EPD-negative lesions were significantly different (*p* = 0.009, Fig. 2d). For all other mpMRI-based parameters, there was no significant correlation with EPD status (ADC_mean_ *p* = 0.546, ADC_min_ *p* = 0.818, and mpMRI volume *p* = 0.162, Table 2).

**Table 2:** Semiquantitative values for PSMA uptake and mpMRI

Semiquantitative parameters	Total	EPD +	EPD−	*p*-value
SUV_max_(mean ± SD)	14.2 ± 10	16.7 ± 10.4	12.4 ± 9.2	0.081
PSMA_vol_(mean ± SD)	6.7 ± 8.4	10.4 ± 10.7	3.9 ± 4.2	**0.004**
PSMA_tot_(mean ± SD)	53.2 ± 82.5	86.7 ± 109.5	28.4 ± 37.1	**0.008**
ADC_min_(mean ± SD)	0.534* ± 0.252	0.525** ± 0.209	0.539 ± 0.276	0.818
ADC_mean_(mean ± SD)	0.942* ± 0.232	0.922** ± .218	0.956 ± 0.238	0.546
mpMRI LCC (mean ± SD)	20.4 ± 16.5	26.6 ± 18.9	15.8 ± 12.6	**0.009**
mpMRI volume (mean ± SD)	3.0 ± 6.0	4.3 ± 8	2.1 ± 3.6	0.162

Statistically significant *p*-values are marked by bold font

*EPD* extraprostatic disease, *SUV* standardized uptake value, *max* maximum, *min* minimum, *SD* standard deviation, *ADC* apparent diffusion coefficient, *LCC* longest capsular contact

*72/73 patients assessable

**30/31 patients assessable

On PSMA scans, both volume-based PET parameters PSMA_tot_ and PSMA_vol_ were significantly higher for lesions with EPD (*p* = 0.008 and *p* = 0.004), while PSMA SUV_max_ did not reach significance (*p* = 0.081) (Table 2, Fig. 2e–f).

### Prediction of ECE based on significant clinical and imaging parameters

**Fig. 3:**
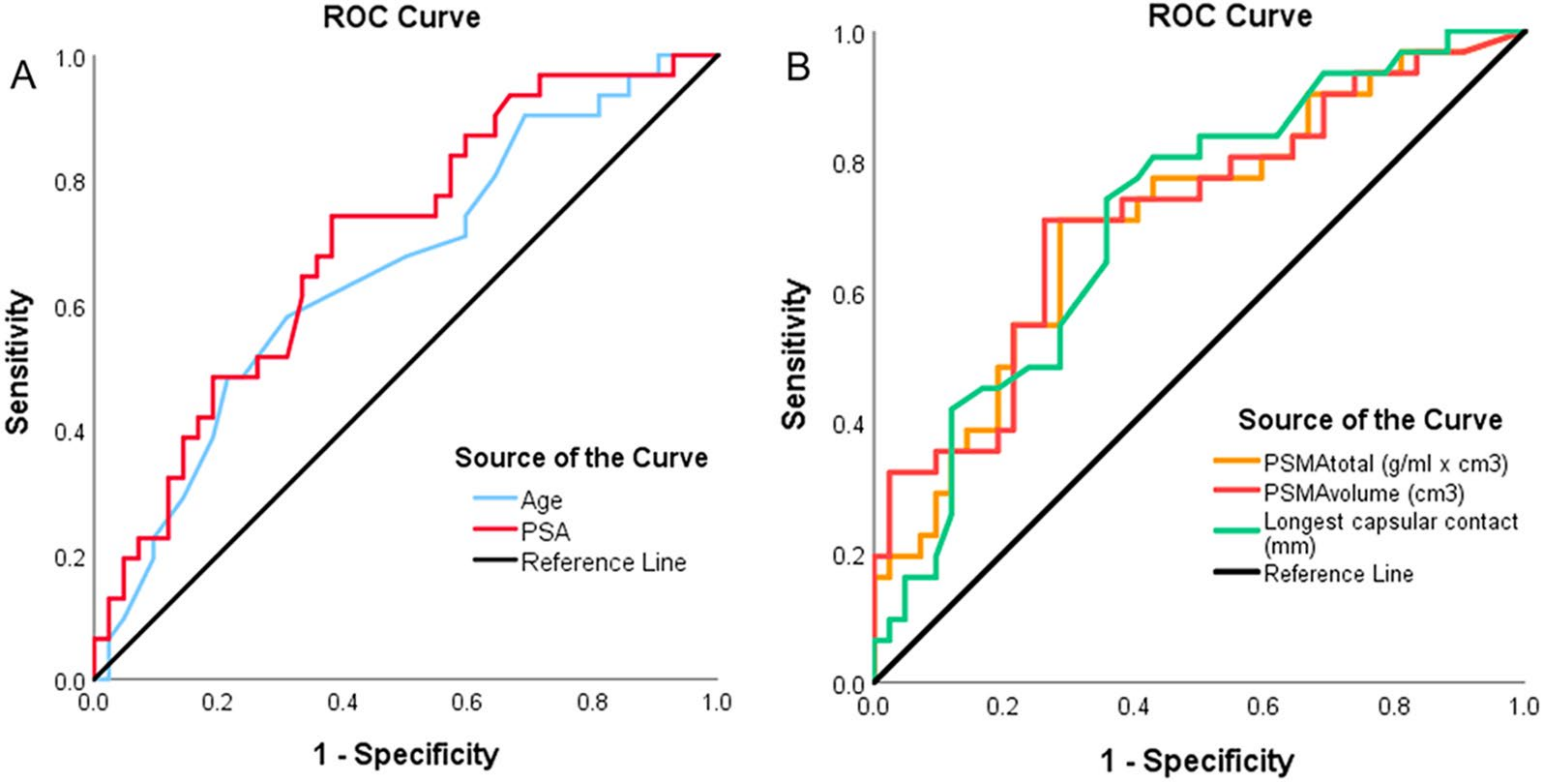
**a** ROC curves analysis for Age and PSA values prediction of EPD. An age of 65.5 y reached an AUC of 0.654, sensitivity of 58.1%, and specificity of 69%. A PSA value of 8.75 ng/ml reached an AUC of 0.7, sensitivity of 74.2%, and specificity of 61.9% **b** ROC curve analysis for PSMA_tot_, PSMA_vol_ and LCC and PSA values prediction of EPD. A PSMA_tot_ of 24.6 g/ml reached an AUC of 0.709, sensitivity of 71%, and specificity of 71.4%. A PSMA_vol_ of 4.41cm^3^ reached an AUC of 0.718, sensitivity of 71%, and specificity of 73.8%. An LCC of 16.5 mm reached an AUC of 0.712, sensitivity of 74.2%, and specificity of 64.3%

The ROC analysis for the two clinical parameters significantly associated with EPD, age and PSA, resulted in an AUC of 0.654 and 0.700, respectively. Using the Youden index, a cutoff at PSA 8.75 ng/ml (Sens 74.2%, Spec 61.9%) and age 65.5 years (Sens 58.1%, Spec 69%) was found (Fig. 3a).

**Fig. 4:**
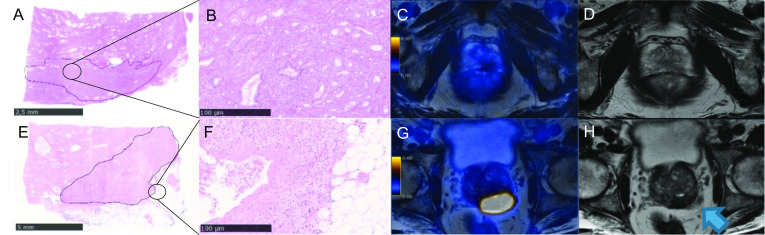
[^68^Ga]PSMA-PET/MRI and mpMRI images of a 69-year-old patient with PSA of 3.7 ng/ml that subsequently underwent radical prostatectomy (ISUP 3). **a**, **b** Histopathology slide negative for EPD with (**a**) overview (bar = 2.5 mm) and (**b**) magnification (bar = 100 µm) showing PCa on the right-posterior mid-gland prostate; **c** corresponding axial [^68^Ga]PSMA PET/MRI with SUV_max_ of 4.7, PSMA_tot_ of 2.0 g/ml, and PSMA_vol_ of 0.485cm3; **d** axial T2-weighted mpMRI showing an LCC of 6 mm. **e**–**h** [^68^Ga]PSMA-PET/MRI images of a 64-year-old patient with PSA of 11 ng/ml that subsequently underwent radical prostatectomy (ISUP 5). **e**–**f** Histopathology slide positive for EPD with (**e**) overview (bar = 5 mm) and (**f**) magnification (bar = 100 µm), showing PCa on the posterior part of the prostatic base (blue arrow); **g** corresponding axial [^68^Ga]PSMA PET/MRI with SUV_max_ of 37.1, PSMA_tot_ of 201.2 g/ml, and PSMA_vol_ of 18.6cm3; **h** axial T2-weighted MRI showing an LCC of 41 mm

**Fig. 5:**
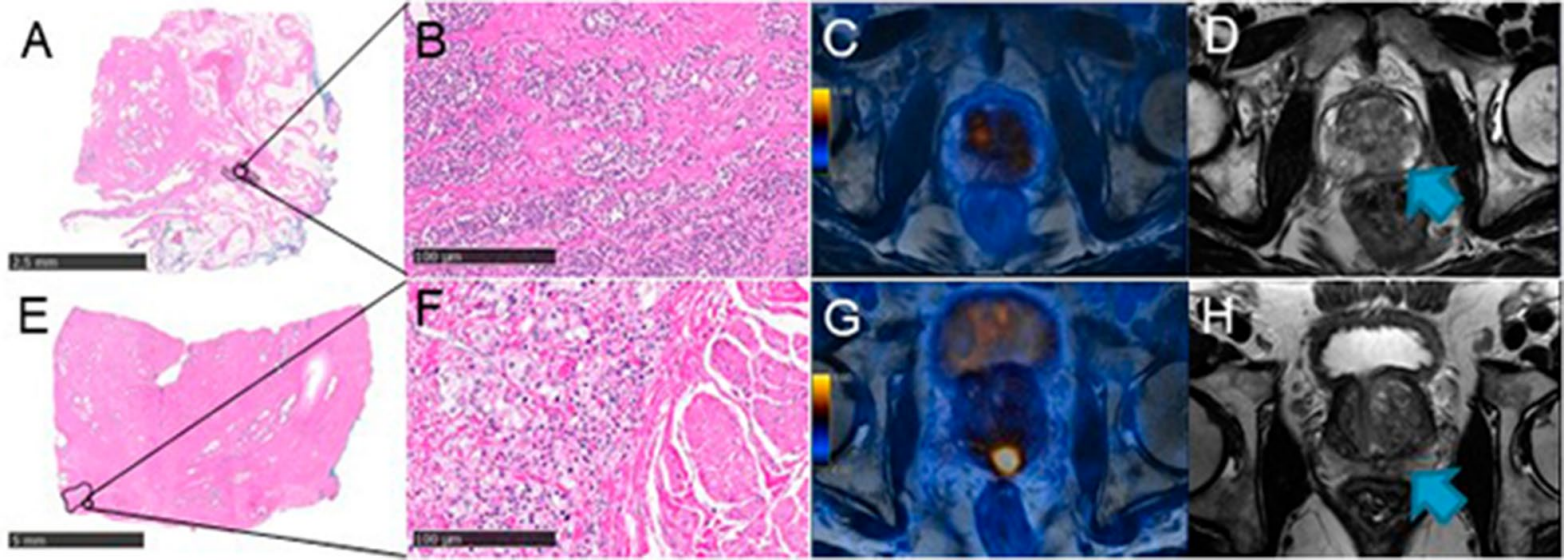
[^68^Ga]PSMA-PET/MRI and mpMRI images of a 66-year-old patient with PSA of 6.1 ng/ml that subsequently underwent radical prostatectomy (ISUP 4). **a**, **b** Histopathology slide positive for EPD with **a** overview (bar = 2.5 mm) and **b** magnification (bar = 100 µm), showing PCa on the posterior left part of the prostate with EPD of 6.5 mm; **c** corresponding axial [^68^Ga]PSMA PET/MRI with SUV_max_ of 4.8, PSMA_tot_ of 9.3 g/ml, and PSMA_vol_ of 2.1cm3; **d** axial T2-weighted mpMRI showing an LCC of 28 mm (blue arrow). **e**–**h** 69-year-old patient with PSA of 5.8 ng/ml that subsequently underwent radical prostatectomy (ISUP 5). **e**–**f** Histopathology slide positive for EPD with **e** overview (bar = 5 mm) and **f** magnification (bar = 100 µm) showing PCa on the posterior mid-gland prostate with a small EPD of 1.5 mm; **g** corresponding axial [^68^Ga]PSMA PET/MRI with SUV_max_ of 11.8, PSMA_tot_ of 36.1 g/ml, and PSMA_vol_ of 6.35cm3; **h** axial T2-weighted mpMRI showing an LCC of 11 mm (blue arrow)

The ROC analysis for the significant imaging parameters LCC, PSMA_tot_, and PSMA_vol_ associated with EPD resulted in an AUC of 0.712, 0.709, and 0.718, respectively. The Youden index resulted in a cutoff for LCC 16.5 mm (Sens 74.2%, Spec 64.3%), PSMA_tot_ 24.6 g/ml × cm^3^ (Sens 71%, Spec 71.4%), and PSMA_vol_ 4.41cm^3^ (Sens 71%, Spec 73.8%), respectively (Fig. 3b). In Fig. 4, we show a concordant negative and positive case, while Fig. 5 illustrates discordant cases regarding imaging parameters prediction of EPD.

### Correlation between imaging parameters and RPE results

**Fig. 6:**
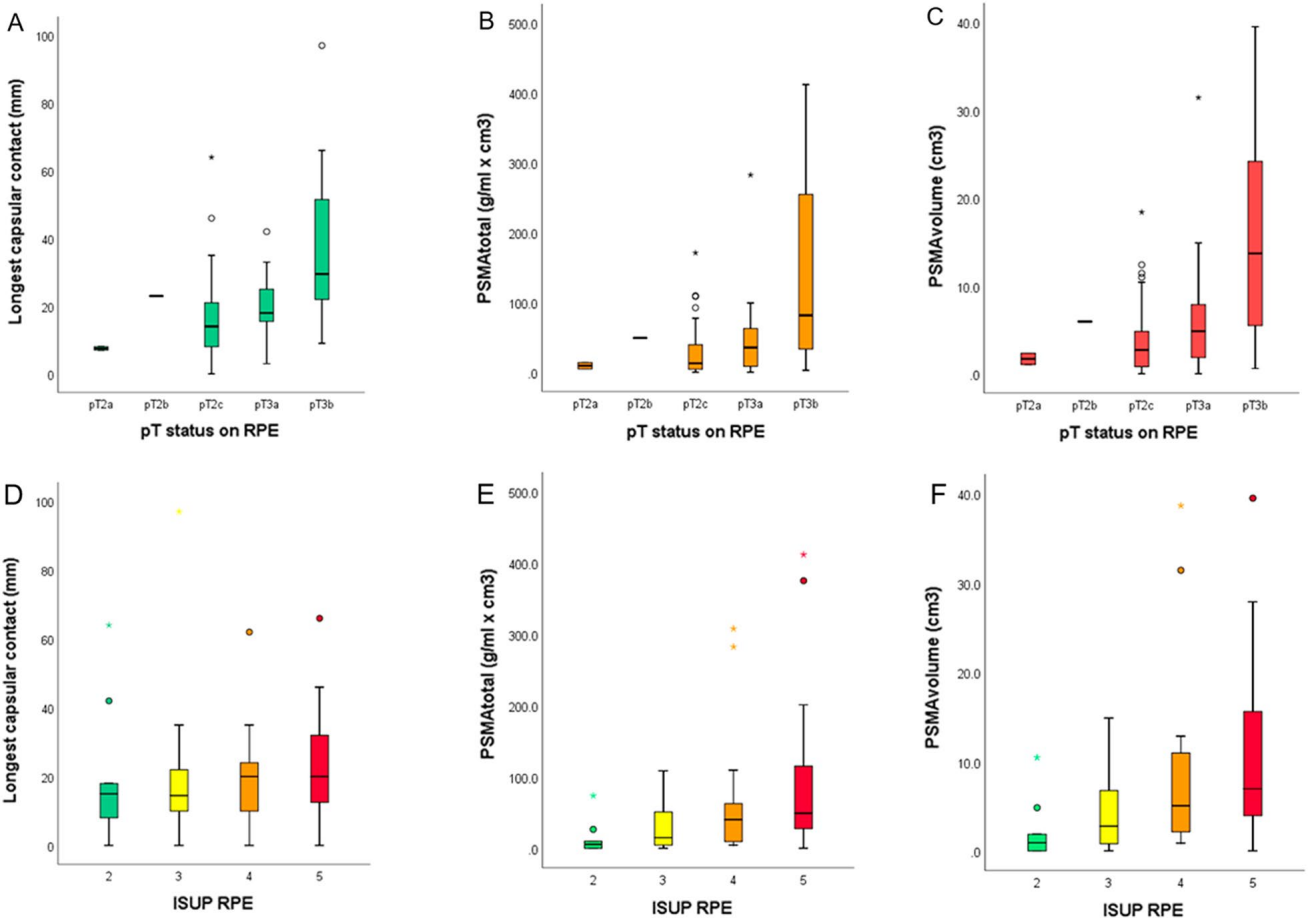
Box plots illustrating the relationship between (**a**) longest capsular contact, (**b**) PSMA_tot_, and (**c**) PSMA_vol_ according to pT status on RPE, and (**d**–**f**) subdivided for ISUP RPE

Overall we observed positive correlation between pT status on RPE and LCC (*r* = 0.420, *p* < 0.001), PSMA_tot_ (*r* = 0.444, *p* < 0.001), and PSMA_vol_ (*r* = 0.471, *p* < 0.001), as shown in Fig. 6a–c. Similarly, we observed an overall positive correlation between ISUP RPE and curvilinear LCC (*r* = 0.119), PSMA_tot_ (*r* = 0.365), and PSMA_vol_ (*r* = 0.355, Fig. 6d–f, Table 3).

**Table 3:** Pearson’s correlation between quantitative parameters and ISUP RPE

Quantitative parameters	ISUP RPE (*n* = 73)	pT on RPE (*n* = 73)
LCC	0.119 (*p* = 0.317)	**0.420 (*p* < 0.001)**
PSMA_tot_	**0.365 (*p* = 0.002)**	**0.444 (*p* < 0.001)**
PSMA_vol_	**0.355 (*p* = 0.02)**	**0.471 (*p* < 0.001)**

Statistically significant *p*-values are marked by bold font

*EPD* extraprostatic disease, *tot* total, *vol* volume, *LCC* longest capsular contact

### Intraclass correlation coefficient

Considering PSMA PET quantitative parameters, excellent agreements were observed for SUV_max_, PSMA_tot_, and PSMA_vol_ with ICC values of 0.996 (95% CI, 0.993–0.997), 0.994 (95% CI, 0.990–0.996), and 0.985 (95% CI, 0.976–0.991), respectively. Moderate-to-good agreement was observed for LCC assessment based on mpMRI, with an ICC of 0.727 (95% CI, 0.565–0.828).

## Discussion

Our results show that quantitative, volume-based PSMA PET parameters are equally predictive for EPD like LCC measured on mpMRI with lower interreader variability. In our cohort, mpMRI curvilinear LCC significantly correlated with EPD (ECE and/or SVI; pT3 status) and reached an AUC of 0.712 (cutoff 16.5 mm, Sens 74.1%, Spec 64.3%). Studies in the literature investigating the diagnostic accuracy of LCC for the detection of EPD generally showed heterogeneous results, with different cutoffs, and a reliable threshold is yet to be established. (PiRADS 2.1 guidelines recommend an arbitrary cutoff of 10 mm [6].) The reason for this variability is likely multifactorial due to different cohorts, methodology (linear versus curvilinear measurements), scanners, and imaging protocols. Using a 1.5 T mpMRI, Dominguez et al. observed that an LCC of 12 mm reached a sensitivity of 69% for EPD prediction [19]; similarly, several authors suggested lower cutoffs for curvilinear LCC compared to our study, reaching higher sensitivities but lower specificities, such as Kongnyuy et al. (3 T MRI) with curvilinear LCC = 12.5 mm (Sens 77%, Spec 59%) [20], Valentin et al. (3 T MRI) with curvilinear LCC = 11.0 mm (Sens 93%, Spec 58%) [21], and Krishna et al. (3 T MRI) with curvilinear LCC > 11.0 mm (Sens 84.9%, Spec 44.8%) [22]. On the other hand, some authors suggested higher cutoffs, such as Baco et al. with a cutoff at curvilinear LCC = 20 mm, reaching an AUC of 0.88 (Sens 79%, Spec 85%) in the prediction of EPD [23]. There is also some controversy regarding the use of linear or curvilinear LCC. Rosenkrantz et al. suggested an optimal cutoff for LCC of 6 mm and 10 mm for EPD and non-focal EPD based on linear measurements, respectively, reaching an AUC of 0.81 even if neither reader had substantial experience in prostate MRI interpretation [24]. Further, Caglic et al. showed that the LCC threshold differs between high- and low-grade tumors, and that a 3D ECE assessment significantly increased sensitivity in diagnosing ECE [25]. Artificial intelligence may be useful for EPD assessment, also from a technical point of view reducing the acquisition time of prostate MRI without reducing image quality or diagnostic performance [26]. Cysouw et al. in a prospective study including 76 staging PCa patients delineated primary tumors using a 50–70% threshold. They obtained 480 radiomics features, and random forest models were trained to predict the presence of EPD, reaching an AUC of 0.76 ± 0.12 (*p* < 0.01) [27]. Also, Cuocolo et al. assessed an MRI radiomics and machine-learning approach (support vector machine) algorithm for EPD identification in 193 PCa patients based on T2 and ADC, with histopathology as the reference standard. The accuracy of the algorithm was similar to expert radiologists’, also using 2 independent external datasets for validation (*p* = 0.39–1). Authors conclude that including their radiomics signature in the EPD grade scoring system may further increase its diagnostic accuracy and reliability in PCa staging, supporting less experienced readers [28].

In the literature, the reported potential of PSMA PET to predict EPD is very heterogeneous [29–31], but a recent meta-analysis of 615 patients came to an averaged sensitivity of 72% and specificity of 87% for extraprostatic extension [32]. Only a few studies assessed the correlation of quantitative PSMA parameters with EPD, mainly regarding SUV_max_ without any cutoff evaluation, such as von Klot et al. observing a significant correlation between SUV_max_ and EPD (*p* = 0.039) in a small PCa cohort (*n* = 21) [33]. This was confirmed by Yilmaz et al. who reported that SUV_max_ and preoperative PSA levels were significantly higher in patients with SVI (*p* < 0.05) [34]. In our cohort, PSMA_tot_ and PSMA_vol_ were significantly higher for lesions with EPD (*p* = 0.008 and *p* = 0.004), while PSMA SUV_max_ did not reach significance (*p* = 0.081). The ROC analysis for PSMA_tot_ and PSMA_vol_ resulted in AUCs of 0.709 and 0.718, respectively. With a cutoff for PSMA_tot_ 24.6 g/ml × cm^3^ and PSMA_vol_ 4.41 cm^3^, a comparable sensitivity and specificity of 71% and 71%/74% could be reached, respectively. Of note, our quantitative assessments of PSMA PET and mpMRI parameters to predict EPD yielded a similar accuracy compared to previously published expert readout-based accuracy of mpMRI (Sens 46%, Spec 75%) and PSMA PET/MRI (Sens 69%, Spec 67%) [13]. Furthermore, we could confirm that PSMA parameters reached an excellent interreader agreement, as it has been already published [35], with a significantly higher ICC > 0.985 (95%CI 0.976–0.997) compared to the only moderate-to-good agreement for LCC quantification on mpMRI (ICC = 0.727, 95%CI 0.565–0.828). These results appear promising even if preliminary, and further multicentric and homogeneous approaches are warranted.

The main limitations of our study were the small sample size and the retrospective nature which may determine a selection bias. Given that our cohort included only intermediate- to high-risk PCa patients, results might not be applicable in cohorts including low-risk PCa patients. However, PSMA PET is generally performed in the analyzed scenario and it is not recommended for staging the low-risk disease. Furthermore, data from mpMRI were not homogeneous (18/73 acquired in external institutions, 25%), with 1.5 T (2/73, 3%), 3 T scanners, and the use of endorectal coils in a few cases (8/73, 11%).

## Conclusion

Quantitative PSMA parameters have a similar potential as mpMRI LCC to predict EPD in patients with intermediate- to high-risk PCa. PSMA PET parameters, however, have a significantly higher interreader agreement.

## Supplementary Information

**Additional file 1**. **Supplemental Table 1**. mpMRI in-house sequences’ details.

## Data Availability

Data are available for bona fide researchers who request it from the authors.

## Abbreviations

ADC: Apparent diffusion coefficient
ECE: Extracapsular extension
EPD: Extraprostatic disease
LCC: Longest capsular contact
mpMRI: Multiparametric magnetic resonance imaging
PCa: Prostate cancer
PET: Positron emission tomography
PSMA: Prostate-specific membrane antigen
RPE: Radical prostatectomy
SVI: Seminal vesicle infiltration
