# Supplementary material for: Quantitative imaging parameters to predict the local staging of prostate cancer in intermediate- to high-risk patients
Source: Insights Imaging. 2022 Apr 15;13:75. doi: 10.1186/s13244-022-01217-4 (PMC9012878; doi:10.1186/s13244-022-01217-4)
Supplement: Supplementary file 1 — Additional file 1. Supplemental Table 1. mpMRI in-house sequences’ details. [file 13244_2022_1217_MOESM1_ESM.docx]

**Supplements**

**Supplemental table1.** mpMRI in-house sequences’ details

|  | Axial DWI EPI (Focus) (Pelvis) | Axial LAVA-FLEX WB (DIXON) | Axial T1w Whole ARC (Pelvis) | Axial T2w FRFSE-XL  (Pelvis) | Coronal T2w WB FRFSE-XL | Coronal T2w  FRFSE-XL  (Pelvis) | Axial DCE  (Lava Dyn)  (Pelvis) | Ax syn. DWI Focus | Sag T2w  FRFSE |
| --- | --- | --- | --- | --- | --- | --- | --- | --- | --- |
| Repetition time, TR (ms) | 4000 | 5·6 | 550 | 5034 | 5538 | 5034 | 6·361 | 3500 | 4678 |
|  |  |  |  |  |  |  |  |  |  |
| Echo time,  TE (ms) | 67·3 | 1·3-2·7 | 8·26 | 120 | 120 | 120 | 2·376 | Minimum | 120 |
|  |  |  |  |  |  |  |  |  |  |
| Flip angle,  FA (degrees) | 90 | 12 | 111 | 140 | 111 | 140 | 30 | - | 140 |
|  |  |  |  |  |  |  |  |  |  |
| Acquisition matrix | 160x80 | 344x 256 | 384x384 | 300x280 | 288x224 | 300x280 | 160x80 | 140x70 | 300x272 |
|  |  |  |  |  |  |  |  |  |  |
| Image size (voxels) | 256x256 | 512x512 | 512x512 | 512x512 | 512x512 | 512x512 | 288x192 | - |  |
|  |  |  |  |  |  |  |  |  |  |
|  |  |  |  |  |  |  |  |  |  |
| Slice thickness (mm) | 4 | 3 | 5 | 3·5 | 5 | 3·5 | 4 | 4 | 3·5 |
|  |  |  |  |  |  |  |  |  |  |
| Signal averages | 8 | 0·68 | 0·5 | 2 | 0·5 | 4 | 0·35 | - |  |
|  |  |  |  |  |  |  |  |  |  |
| b-values (s/mm2) and signal averages | 0 (6 av.)  400 (8 av.)  700 (16 av.) |  |  |  |  |  |  | 0  400  1000  1500  2000 |  |
|  |  |  |  |  |  |  |  |  |  |
| Diffusion direction | ‘All’ |  |  |  |  |  |  | All |  |
|  |  |  |  |  |  |  |  |  |  |
| Bandwidth (Hz/pixel) | 1953 | 166 | 62·5 | 50 | 90·9 | 50 | 62·5 | 250 | 50 |
|  |  |  |  |  |  |  |  |  |  |
| Acquisition time (mm:ss) | 5:41 | 0:18 | 1:44 | 3:27 | 0:50 | 3:27 | 3:27 | 4:05 | 3:12 |
